# An abrupt decrease in arterial blood pressure may predict a high level carbon dioxide embolism in retroperitoneoscopic surgery: case report and a literature review

**DOI:** 10.1186/s12894-023-01192-y

**Published:** 2023-03-07

**Authors:** Jianwei Wang, Zhengqing Bao, Libo Man

**Affiliations:** Department of Urology, Beijing Jishuitan Hospital, No. 31 Xinjiekou East Street, Xicheng District, Beijing, 100035 China

**Keywords:** Case report, Abrupt decrease, Arterial blood pressure, Carbon dioxide embolism, Retroperitoneoscopic surgery

## Abstract

**Background:**

Carbon dioxide (CO_2_) embolism is the primary suspect in most cases of intraoperative “cardiovascular” collapse. However, there are few reports about CO_2_ embolism in retroperitoneal laparoscopy.

**Case presentation:**

An abrupt decrease in arterial blood pressure was noted in time of retroperitoneoscopic adrenalectomy in a 40 years old male patient with adrenal adenoma. The end-tidal carbon dioxide (EtCO_2_) and saturation of oxygen were stable with normal cardiography until anesthesiologists found the change of resistant of peripheral circulation, then they gave us a hint of hemorrhage. However, the blood pressure had no reaction to one bolus of epinephrine administration when trying to improve the circulation. Five minutes later, a sudden fall of blood pressure was noted, and then we stopped the processing of cutting tissue and trying to coagulate any bleeding in the operation field. Further vasopressor support proved to be completely ineffective. With the help of transesophageal echocardiography, we found the bubbles in the right atrium, which confirmed the diagnosis of an intraoperative gas embolism (Grade IV). We stopped the carbon dioxide insufflation and deflated the retroperitoneal cavity. All the bubbles in the right atrium totally disappeared and the blood pressure, resistance of peripheral circulation and cardiac output returned to normal 20 min later. We continued the operation and completed it in 40 min with the 10 mmHg air pressure.

**Conclusion:**

CO_2_ embolism may occour during retroperitoneoscopic adrenalectomy, and an acute decrease in arterial blood pressure should alert both the urologists and anesthesiologists to this rare and fatal complication.

## Background

Laparoscopic surgeries by transperitoneal, retroperitoneal approach and robotic assisted laparoscopy have gained increasing popularity and rapidly expanded as minimal invasive techniques. One of the fatal complications is carbon dioxide (CO_2_) embolism due to the direct puncture of the major vessels, and other courses of CO_2_ embolism, such as gas absorption through the vein plexus, need further exploration [1, 2].

A multicenter research of incidents in time of laparoscopic surgery indicated that CO_2_ embolism is the primary suspect in most cases of intraoperative “cardiovascular” collapse [3]. So, by monitoring the hemodynamic and respiratory variables, early detection of CO_2_ embolism followed by immediate treatment may avoid serious complications and decrease the mortality caused by clinically significant CO_2_ embolism.

## Case presentation

A 40 years old Chinese male presented with a 3-month history of weakness of lower extremities. His family and past medical history were unremarkable. On admission, his blood pressure was 165/95 mmHg and the blood potassium is 2.0 mmol/L. The further endocrinal examinations confirmed the diagnosis of primary aldosteronism (PA) and computed tomography (CT) scan showed a radiolucent nodule of 2.3 cm, with low density on unenhanced CT and low vascularity on contrast-enhanced CT, at the tip of the right adrenal gland. Thus, we diagnosed a right adrenal hypersecretion of aldosterone.

The patient underwent right retroperitoneoscopic adrenalectomy under general anesthesia in the operating room. The end-tidal CO_2_ (EtCO_2_) was maintained at 35–45 mmHg throughout the surgery. ECG and invasive arterial blood gas analysis were conducted. After insertion of the internal jugular central venous catheter, the patient was positioned in the lateral position with the right side up, then the table flexed to accentuate the left flank.

**Fig. 1:**
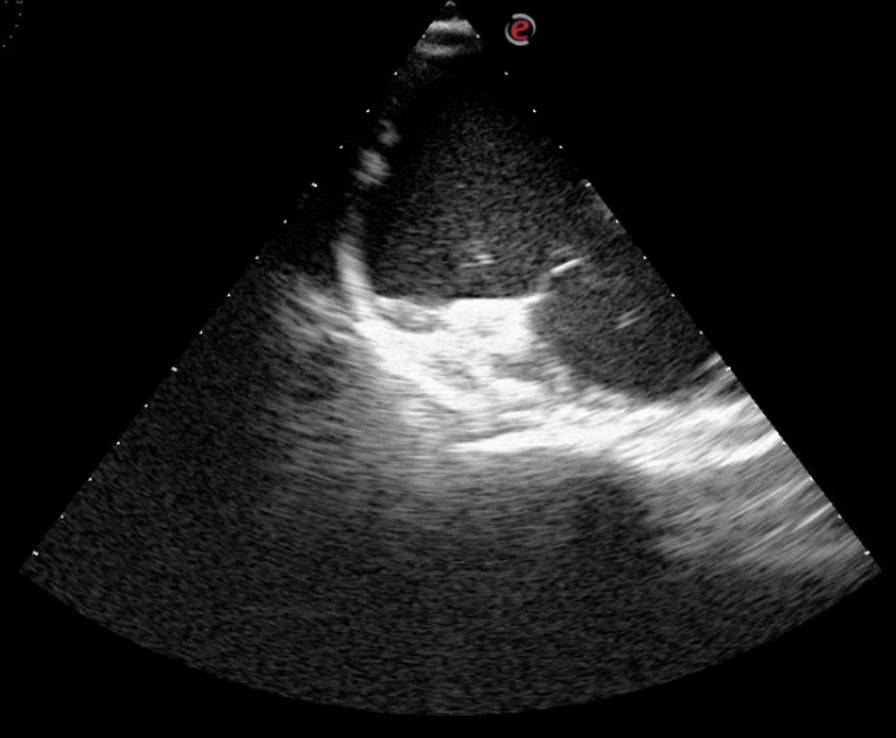
CO_2_ embolism in the right atrium

The retroperitoneal pressure was maintained at 14 mmHg using a CO_2_ insufflator. And the patients remained hemodynamic stable when we were building the retroperitoneal cavity and removing the retroperitoneal fat tissue. About 20 min into the surgery, when finishing the dissection of the inferior border of the gland after opening the Gerotar’s fascia, the anesthesiologists found an abrupt decrease in arterial blood pressure (ABP) and change of resistant of peripheral circulation, then warned us about the possibility of an ongoing hemorrahage. We checked and made sure the clearness of the operation field, then went on the operating. Five minutes later, a sudden fall of blood pressure was noted and the blood pressure had no reaction after one bolus of epinephrine administration when trying to improve the circulation. We stopped the processing of cutting tissue and trying to coagulate any bleeding in the operation field. Further vasopressor support proved to be completely ineffective. With the help of TEE, we found the bubbles almost fully filled in the right atrium, which confirmed the diagnosis of an intraoperative gas embolism (Fig. 1). According to classification system made by Schmandra [4], it was a Grade IV carbon dioxide embolism.

We stopped the carbon dioxide insufflation and deflated the retroperitoneal cavity. All the bubbles in the right atrium totally disappeared and the blood pressure, resistance of peripheral circulation and cardiac output returned to normal after 20 min later. Intraoperatively, no change of EtCO_2_ was observed and it was accompanied by stable saturation of oxygen and normal cardiography.

Finally, we finish the operation in 40 min with the 10 mmHg air pressure. Although, casually, the Grade I–II embolisms were observed by TEE during the following process, all monitoring parameters were stable and the anesthesiologist permitted the continuation of our operation till the end of our procedure.

## Discussion

Compared to open procedures, laparoscopic surgeries have been extensively used for its substantially low morbidity and mortality. Additionally, retroperitoneoscopy is an unusual but useful approach for adrenal surgery. Many surgeons are not confident with this anatomical region and its exploration is related with several complications[5, 6]. CO_2_ embolism is one of the life-threatening complications that is related to pneumoperitoneum occurrence and is rare in the conventional open approach surgery. Although clinically significant CO_2_ embolism is not a common complication, it’s associated with certain mortality of 50% [7].

During the operation, the patient may show electrocardiogram changes or hemodynamic instability at the moment of venous gas embolism occurrence [8]. EtCO_2_ monitoring has been regarded as a noninvasive and sensitive method of detecting gas embolism[2, 4, 9]. Previous animal modal experiments have indicated that the initial response to CO_2_ embolism was a EtCO_2_ decrease [9]. However, a sudden increase in EtCO_2_ (from 35 to 58 mmHg) followed by an abrupt fall (to 18 mmHg) was found during retroperitoneoscopic adrenalectomy in a 23 years old patient diagnosed with adrenal hyperplasia [2].

Not all the patients show electrocardiogram changes or hemodynamic instability when CO_2_ embolism occurs [8]. And the early change we detected in this case is the fall of the cardiac output and there is no change of EtCO_2_ throughout the whole procedure. So, monitoring the change of EtCO_2_ under general anesthesia is not always reliable if we want to detect the occurrence of CO_2_ embolism. Although the changes in EtCO_2_ may be precipitous and of short duration, and easy to be missed in the operation room [10], the most important is insufflation should immediately be stopped if a rapid change in EtCO_2_ occurs [11].

TEE was thought to be the most sensitive means for detecting intravenous injection of CO_2_ as small as 0.1 mL/kg [9], and regarded as the gold standard method for diagnosing gas embolism [8, 9, 12]. In the era without TEE, the incidence of CO_2_ embolism during laparoscopic surgery was reported very low, ranging from 0.001 to 0.59% [2, 13, 14]. TEE can project the incidence of CO_2_ embolism as high as 100% in specific types of laparoscopic procedures [10]. however, it was complained by someone who insisted that TEE is too sensitive and may pick up small emboli that have no importance [11]. Schmandra [4] classified the CO_2_ emboli detected by 2-dimensional transesophageal echocardiography as five grades. So, this case we report is the Grade IV CO_2_ embolism according to this classification, that is the most serious situation and may have the highest risk of the mortality.

CO_2_ embolism can be manifested by itself through a gas lock effect, and the mixture of blood and gas by cardiac contractions can induce a foam that influence on pulmonary vessels [11]. Abrupt pulmonary hypertension ensues and leads rapidly to fatal right ventricular failure. So, a continuous decrease in venous return compromises global cardiac output and ends in hemodynamic instability and cardiac collapse [1, 2, 15]. In addition, the longer retention of bubbles in the right ventricle outflow tract leads to the reduced cardiac output [16]. The early incidents are usually inadvertent injection of CO2 into a large vein, artery or solid organ especially by the veress needle or trocar [1, 8, 11, 16] and usually occur during or shortly after insufflation of carbon dioxide during surgery. Also, lethal gas embolism had been reported in the absence of visible lesion to blood vessels [1, 17], the presumed mechanism of CO_2_ embolism is the force of pressured CO_2_ through venous vessles [1, 2, 8]. So the CO_2_ may be entrained into circulation through an opening in the abdominal wall either in injured blood vessel or at the site of surgery, and the highly soluble in blood makes carbon dioxide can be rapidly absorpted into the blood scream across the peritoneum [18].

Patients who had previously undergone abdominal surgery would experience the CO_2_ embolism or even pulmonary edema due to ruptured peritoneal adhesions, which caused CO_2_ to enter into the circulation system through the injured vein. For those patients undergoing transperitoneal laparoscopy insufflation should be begun with a low gas pressure and a slow flow rate, which were necessary to limit the gas volume embolized in the event of venous cannulation inadvertently [11].

Unlike transperitoneal laparoscopy, retroperitoneal technique needs an artificial space supported by the continuous insufflating CO_2_ with certain pressure, that forces the direct absorption of CO_2_ into venous plexus, too [2]. Also, the retroperitoneal space is non-expansible and fairly limited, increasing the risk of accidental damage in time of endoscopic surgery including damage to small vessels.

## Conclusion

CO_2_ embolism may occour during retroperitoneoscopic adrenalectomy, and an acute decrease in arterial blood pressure should alert both the urologists and anesthesiologists to this rare and fatal complication.

## Data Availability

The data used in this study are available from the corresponding author upon reasonable request.

## Abbreviations

CO_2_: Carbon dioxide
ABP: Arterial blood pressure
EtCO_2_: End-tidal carbon dioxide
TEE: Transesophageal echocardiography
PA: Primary aldosteronism
CT: Computed tomography
